# Frequency and Duration of SARS-CoV-2 Shedding in Oral Fluid Samples Assessed by a Modified Commercial Rapid Molecular Assay

**DOI:** 10.3390/v12101184

**Published:** 2020-10-20

**Authors:** Licia Bordi, Giuseppe Sberna, Eleonora Lalle, Pierluca Piselli, Francesca Colavita, Emanuele Nicastri, Andrea Antinori, Evangelo Boumis, Nicola Petrosillo, Luisa Marchioni, Giulia Minnucci, Elena D’Agostini, Concetta Castilletti, Franco Locatelli, Alimuddin Zumla, Giuseppe Ippolito, Maria Rosaria Capobianchi, on behalf of INMI ReCOVeRI Study Group

**Affiliations:** 1 Laboratory of Virology, National Institute for Infectious Diseases “L. Spallanzani” IRCCS, 00149 Rome, Italy; giuseppe.sberna@inmi.it (G.S.); eleonora.lalle@inmi.it (E.L.); francesca.colavita@inmi.it (F.C.); concetta.castilletti@inmi.it (C.C.); maria.capobianchi@inmi.it (M.R.C.); 2 Epidemiology Department, National Institute for Infectious Diseases “L. Spallanzani” IRCCS, 00149 Rome, Italy; pierluca.piselli@inmi.it; 3 Clinical Department, National Institute for Infectious Diseases “L. Spallanzani” IRCCS, 00149 Rome, Italy; emanuele.nicastri@inmi.it (E.N.); andrea.antinori@inmi.it (A.A.); evangelo.boumis@inmi.it (E.B.); nicola.petrosillo@inmi.it (N.P.); luisa.marchioni@inmi.it (L.M.); 4 R&D Department, DiaSorin Molecular LLC, Cypress, CA 90630, USA; giulia.minnucci@diasorin.it (G.M.); elena.dagostini@diasorin.it (E.D.); 5 Department of Pediatric Hematology and Oncology, IRCCS Ospedale Pediatrico Bambino Gesù, 00165 Rome, Italy; franco.locatelli@opbg.net; 6 Division of Infection and Immunity, University College London, London WC1E 6BT, UK; a.zumla@ucl.ac.uk; 7 NIHR Biomedical Research Centre, UCL Hospitals NHS Foundation Trust, London W1T 7DN, UK; 8 Scientific Direction, National Institute for Infectious Diseases “L. Spallanzani” IRCCS, 00149 Rome, Italy; giuseppe.ippolito@inmi.it

**Keywords:** SARS-CoV-2 RNA, oral fluid, real time RT-PCR, direct assay

## Abstract

Background: RT-PCR on nasopharyngeal (NPS)/oropharyngeal swabs is the gold standard for diagnosis of SARS-CoV-2 infection and viral load monitoring. Oral fluid (OF) is an alternate clinical sample, easy and safer to collect and could be useful for COVID-19 diagnosis, monitoring viral load and shedding. Methods: Optimal assay conditions and analytical sensitivity were established for the commercial Simplexa™ COVID-19 Direct assay adapted to OF matrix. The assay was used to test 337 OF and NPS specimens collected in parallel from 164 hospitalized patients; 50 bronchoalveolar lavage (BAL) specimens from a subgroup of severe COVID-19 cases were also analysed. Results: Using Simplexa™ COVID-19 Direct on OF matrix, 100% analytical detection down to 1 TCID50/mL (corresponding to 4 × 10^3^ copies (cp)/mL) was observed. No crossreaction with other viruses transmitted through the respiratory toute was observed. Parallel testing of 337 OF and NPS samples showed highly concordant results (κ = 0.831; 95 % CI = 0.771–0.891), and high correlation of Ct values (r = 0.921; *p* < 0.0001). High concordance and elevated correlation was observed also between OF and BAL. Prolonged viral RNA shedding was observed up to 100 days from symptoms onset (DSO), with 32% and 29% positivity observed in OF and NPS samples, respectively, collected between 60 and 100 DSO. Conclusions: Simplexa™ COVID-19 Direct assays on OF have high sensitivity and specificity to detect SARS-CoV-2 RNA and provide an alternative to NPS for diagnosis and monitoring SARS-CoV-2 shedding.

## 1. Introduction

Since the global pandemic spread of SARS-CoV-2 [1,2,3], a priority focus has been on development of rapid and sensitive diagnostic assays using easily obtainable clinical samples. Diagnostic testing for SARS-CoV-2 infection by viral RNA detection in respiratory specimens is required for decision making for clinical management, infection control or public health measures, triage and isolation in healthcare facilities. The WHO currently recommends RT-PCR testing using nasopharyngeal (NPS) and oropharyngeal swabs (OPS) as gold standard for SARS-CoV-2 diagnosis and for monitoring viral load [4,5]. OF has been suggested as an alternate clinical sample, easy and safer to collect, minimizing exposure of healthcare workers and could be useful for making a diagnosis and measuring SARS-CoV-2 viral load and viral shedding during the course of the illness and convalescence [6,7,8,9,10,11,12,13]. To et al., demonstrated that SARS-CoV-2 was present in OF specimen of 11 out of 12 patients, with viral load being higher during the first week after symptoms onset and declining thereafter, being detectable until 25 days after symptoms onset (DSO) [14,15]. In another study, SARS-CoV-2 RNA was detected in OF of one patient for prolonged period, up to 37 DSO [16]. We evaluated the use of commercial Simplexa™ COVID-19 Direct assay on OF samples from hospitalized COVID-19 patients, for identification of SARS-CoV-2 RNA, duration of viral shedding, and determining the assay specificity and sensitivity on OF samples compared to NPS and BAL samples.

## 2. Materials and Methods

### 2.1. Patients and Clinical Specimens Collection

OF specimens were collected from 164 patients hospitalized at National Institute for Infectious Diseases “Lazzaro Spallanzani” (INMI) in Rome. The median age of patients was 59 years (IQR: 43–73), 111 males (67.6%) and 53 females (32.3%). A total of 337 of OF samples were collected in parallel with NPS and results were compared; 50 BAL samples were also collected and analysed concomitantly with NPS and OF samples from a subgroup of patients attending the Intensive Care Unit, showing more severe presentation (PaO2/FiO2 < 100), 7 of whom subsequently died. In this study, patients admitted with suspect of COVID-19 but with definitive diagnosis other than COVID-19, were considered as negative controls. Number of patients and clinical characteristics are described in Table 1.

NPS were immediately put into sterile tubes containing 2–3 mL of viral transport media, like COPAN UTM^®^ Universal Transport Medium; from a subgroup of patients with more severe manifestations also BAL samples were collected. As far as OF is concerned, most specimens were collected by passive drooling, spontaneusly produced without external stimuli; for some patients, to obviate the scarce salivation, sublingual OF was collected using sterile pipettes. All OF were collected neat, without any type of diluent and at least 30 min after drinking or eating or washing theeth.

### 2.2. Analytic Sensitivity

The SARS-CoV-2 isolate 2019-nCoV/Italy-INMI1 [17] was propagated in Vero E6 cells (C1008; African green monkey kidney cells). Cells were maintained in Dulbecco’s minimal essential medium (DMEM) containing 10% foetal bovine serum (FBS) and 0∙05 mg/mL gentamycin at 37 °C with 5% CO2 and FBS concentration was reduced to 2% for viral propagation.

The infectious titre of the viral stock used in the study, performed by Reed and Muench method on VeroE6 cells, was 10^7^ TCDI50/mL. The evaluation of the corresponding concentration of RNA copies (cp)/mL in the viral stock preparation was performed as follows: SARS-CoV-2 RNA was extracted from the isolate and amplified by real-time quantitative RT-PCR (qRT-PCR) in Rotor-GeneQ Real-Time cycler (Qiagen, Hilden, Germany) using RealStar^®^ SARS-CoV-2 RT-PCR Kit 1.0 (Altona Diagnostic GmbH, Hamburg, Germany). A standard curve prepared through serial dilutions of Corman’s E-SARS-CoV-2 gene [18], obtained by European Virus Archive – GLOBALEVAg has been used to determine the concentration of the virus stock, corresponding to 10^10^ RNA cp/mL.

To establish the analytical sensitivity, SARS-CoV-2 particles from the viral stock were spiked into a pool of OF coming from 25 healthy donors, mixed together and diluted 1:1 with 0.9% NaCl isotonic solution. Serial ten-fold dilutions from 10^7^ to 10^−3^ TCDI50/mL were prepared to be tested in triplicates. When established the last dilution with 100% of positive results, obtained at 1 TCID50/mL, five replicates of serial 1:2 dilutions were performed until reaching 0.025 TCID50/mL. The results were used to obtain the limit of detection (LOD) by Probit analysis.

### 2.3. Analytic Specificity

To assess analytical specificity, OF from 5 healthy donors were mixed together, diluted 1:1 with 0.9% NaCl isotonic solution, aliquoted in different tubes; each tube was spiked with different respiratory viruses and loaded on MDX instrument. The following viral stocks were used: Measles virus, Edmonton strain, Titer 10^4.53^ TCID50/mL; Influenza B virus, B/Shandong/7/97 strain, HA titer 1:640; Influenza A H3N2 virus, A/Pt. Chalmers/1/73 strain, HA titer 1:320; Adenovirus 5, Adenoid 75 strain, titer 10^7.75^ TCID50/mL; Human coronavirus OC43, Pt isolate, titer 10^6.54^ TCID50/mL; Human coronavirus 229E, Pt isolate, titer 10^5.37^ TCID50/mL.

### 2.4. Simplexa™ COVID-19 Direct Assay

Simplexa™ COVID-19 direct assay is a real-time RT-PCR system that enables the direct amplification of Coronavirus SARS-CoV-2 RNA from several specimens, without sample processing like RNA extraction. In the Simplexa™ COVID-19 Direct assay (DiaSorin Molecular LLC, Cypress, CA 90630, U.S.A.), fluorescent probes are used together with corresponding forward and reverse primers to amplify two different regions of the SARS-CoV-2 genome: *ORF1ab* and *S* gene; an RNA internal control is used to detect RT-PCR failure and/or inhibition. For testing with the Simplexa™ COVID-19 Direct assay, one vial of Reaction Mix was thawed for each sample followed by loading 50 μL of sample (OF) that was previously diluted 1:1 with 0.9% NaCl and 50 μL of Reaction Mix to their specific wells on a direct amplification disk (DAD). The DAD was then loaded onto the LIAISON^®^ MDX instrument (DiaSorin Molecular), which is a compact and expandable thermal cycler with an extremely small footprint and the capability to connect up to four instruments with a single laptop. Upon completion of the run, the software automatically calculates and provides easy to understand results with the ability to check amplification curves after a run. Samples with Ct values < 40 were considered positive, according to test procedure indications; for statistical calculations, an arbitrary value of 45 Ct was assigned to negative samples.

### 2.5. Statistical Analysis

Data management and analyses were performed using IBM SPSS Statistics version 26 (IBM Corp., Armonk, NY, USA), STATA version 15 (Stata Corp LP, College Station, TX, USA) or GraphPad Prism version 8.00 (GraphPad Software, La Jolla, CA, USA). Descriptive analysis was performed to characterize patients enrolled in the study and above described. Median values and interquartile ranges (IQR) were used to describe numerical variables, while counts and percentages were employed for categorical variables. The analytical sensitivity (SARS-Cov-2 copy number and TCDI50 at a 95% detection proportion) was calculated by probit analysis, using the MedCalc statistical software (MedCalc Software Ltd., Ostend, Belgium), on the basis of results obtained by several replicates of serial dilutions of the 2019-nCoV/Italy-INMI1 spiked into OF matrix. The evaluation of the qualitative concordance between results was performed using the weighted Cohen Kappa statistics [19] and its 95% confidence interval (CI); agreement was evaluated as: poor (less than 0.50), moderate (0.50–0.74), substantial (0.75–0.90), and almost perfect if greater than 0.90. Linear regression analysis, adjusted for gender and age, was used to evaluate the relationship between the two quantitative results. To account for the possible correlation, that may arise from multiple samples belonging to the same patient, robust standard errors were computed.

### 2.6. Ethical statement

This work can be considered exempt from continuous review by an institutional ethical review board, because it comprises secondary use of completely anonymized specimens.

## 3. Results

### 3.1. Analytical Sensitivity and Lower Limit of Detection (LOD)

**Figure 1:**
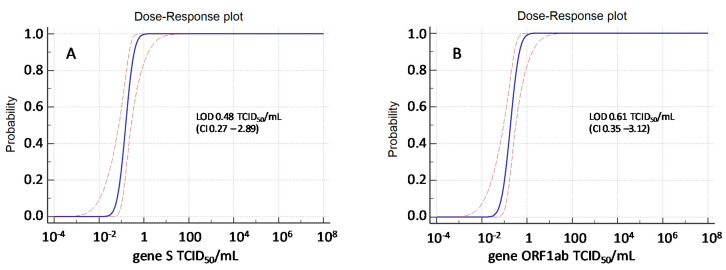
Probit analysis of Simplexa Covid-19 Direct Assay applied to OF samples spiked with virus preparation both for S gene (**A**) and ORF1ab gene (**B**).

The analytical sensitivity (i.e., the limit of detection, LOD, corresponding to the concentration of SARS-CoV-2-RNA detected with response probability of 95% for either *S* or *ORF1ab*) was determined by Probit regression model, and resulted to be 0.48 (CI: 0.27–2.89) TCID50/mL for *S* and 0.61 (CI: 0.35–3.01) TCID50/mL for *ORF1ab*, corresponding to 3.28 (CI 3.03–4.06) logRNA cp/mL and 3.38 (CI 3.14–4.09) logRNA cp/mL, respectively (Figure 1). Analytical sensitivity was similar to that obtained for NPS in a previous study from our group, using the same virus isolate and the same experimental methods, being 0.40 (CI: 0.2–1.5) TCID50/mL for *S* and 0.40 (CI: 0.2–1.3) TCID50/mL for *ORF1ab* corresponding to 3.2 (CI: 2.9–3.8) log10 cp/mL and 3.2 log10 (CI: 2.9–3.7) log10 cp/mL for *S* and *ORF1ab*, respectively (around 1500 cp/mL) [20]. However, a similar analytical sensitivity (1200 cp/mL) was also described for NPS in two additional assays widely used in SARS-CoV-2 molecular diagnosis: RealStar^®^ SARS-CoV-2 RT-PCR Kit 1.0 (Altona Diagnostics, Hamburg, Germany: https://www.fda.gov/media/137252/download) and CDC COVID-19 RT-PCR panel assay (IDT, Coralville, IA: https://www.fda.gov/media/134922/download) [21].

### 3.2. Analytic Specificity

Results obtained for OF spiked with H-CoV229E, H-CoV OC43, ADV, FluA; FluB and MV confirmed high specificity, and lack cross-reactivity with other viruses transmitted through the respiratory toute.

### 3.3. Performance Evaluation on Clinical Specimens

**Figure 2:**
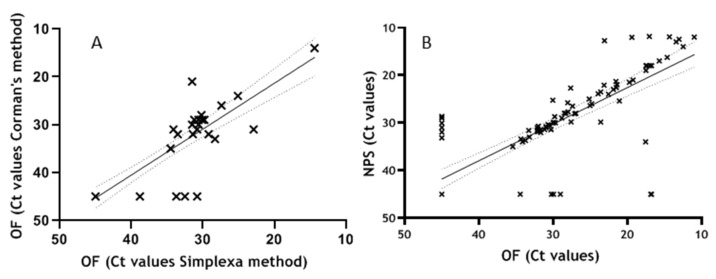
(**A**) Correlation between Simplexa™ COVID-19 Direct assay and reference method applied to OF samples. Ct values obtained on 41 OF samples tested in parallel with Simplexa™ COVID-19 Direct assay and RT-PCR by Corman VM reference method are included in the linear regression analysis (r = 0.770; *p* < 0.0001). (**B**) Correlation between NPS and OF samples. Results obtained from 292 samples tested for the presence of SARS-CoV-2 RNA both in NPS and OF matrices are included in linear regression adjusted for cluster, sex and age (r = 0.921; *p* < 0.0001).

The first performance evaluation on clinical specimen was done by testing 41 consecutive OF samples, including 9 samples from SARS-CoV-2-negative patients, with the Simplexa™ COVID-19 Direct assay and comparing results with that obtained using RT-PCR method established by Corman VM. et al. [18] as reference assay. Analysis showed a substantial concordance in SARS-CoV-2 RNA detection between the two assays (κ = 0.8; 95% CI = 0.612–0.982). Of the 41 samples, 21 resulted positive to both tests, 4 resulted positive only using Simplexa and 16 resulted negative to both assays. Notably, the 4 discordant results positive with Simplexa™ COVID-19 Direct assay but negative with Corman’s method, came from patients with clinically confirmed COVID-19. The Ct values obtained in the two assays show good correlation (r = 0.770; *p* < 0.0001) in linear regression analysis, Figure 2A.

The performance of Simplexa™ COVID-19 Direct assays on clinical specimens was further established by testing in parallel NPS and OF samples for the presence of SARS-CoV-2 RNA. Concordance analyses performed on 337 samples, showed a total of 309 concordant and 28 discordant results, with κ = 0.831; 95 % CI = 0.771–0.891. Linear regression analysis adjusted for cluster of repeated measures, sex and age, performed on 292 samples for which Ct values were available for both matrices, showed elevated correlation of Ct values among NPS and OF (r = 0.921; *p* < 0.0001)(Figure 2B). An even higher correlation was obtained excluding repeated measures and considering only first results from each patient (r = 0.958 and *p* < 0.0001), thus confirming high correlation of Ct values among NPS and OF.

### 3.4. Frequency and Duration of SARS-CoV-2 Shedding

**Figure 3:**
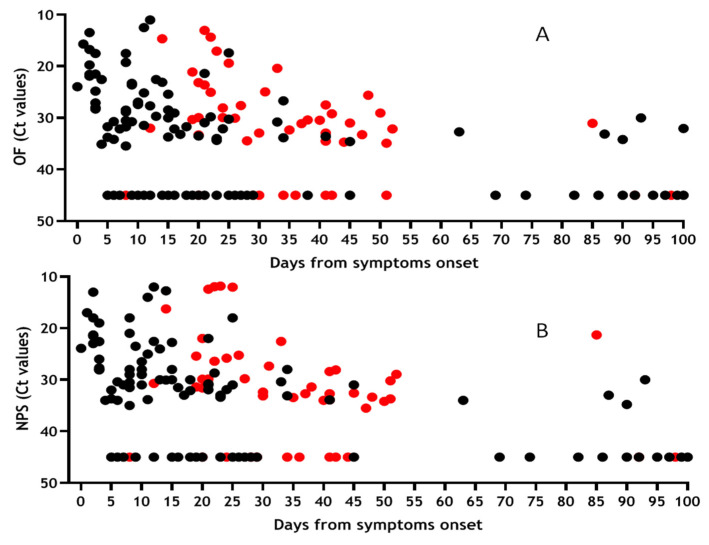
Shedding of SARS-CoV-2 RNA in OF and NPS samples based on days from symptom onset. Results obtained from 162 samples tested for the presence of SARS-CoV-2 RNA both in OF (**A**) and NPS (**B**) and expressed as Ct values vs. days from symptoms onset. Red symbols refer to samples coming from patients with severe COVID-19 disease, while black symbols refer to paucisymptomatic + asymptomatic patients.

For 162 samples we were able to analyse the presence of viral RNA both in OF (Figure 3A) and NPS (Figure 3B) samples, considering the days from symptoms onset (DSO). For asymptomatic individuals, DSO has been calculated from the time of notification to the surveillance system. Presence of RNA in both matrices was observed during the first 30 DSO (67% OF; 72% NPS), remaining stable between 30 and 60 DSO with similar frequency (65% OF; 76% NPS) and was still observed until 100 DSO (32% OF; 29% NPS). Among the analysed samples, 50 were from severe patients (red symbols) while the remaing samples were from paucisymptomatic and asympomatic patients (black symbols).

Moreover, statistical comparison between OF and NPS has been performed, showing no significant difference (*p* > 0.05) between median Ct values in OF and NPS, neither in total nor according to different DSO intervals (Table 2).

Results obtained from statistical analysis confirmed a comparable trend in the two matrices OF and NPS, with Ct median values lower in the first 30 DSO (corresponding to higher viral load) progressively increasing at 60 and > 60 DSO, as expected.

Concerning gender, median Ct values in both district were slightly higher in males (median Ct in OF: 33.5; median Ct in NPS: 32.9) than in females (median Ct in OF: 31,4; median Ct in NPS: 30.5), despite the differences were not significant. Concerning age, Ct values both in OF and NPS were significantly lower in patients <60 years (i.e., according to the median value of patient’s age) (Table 3).

**Figure 4:**
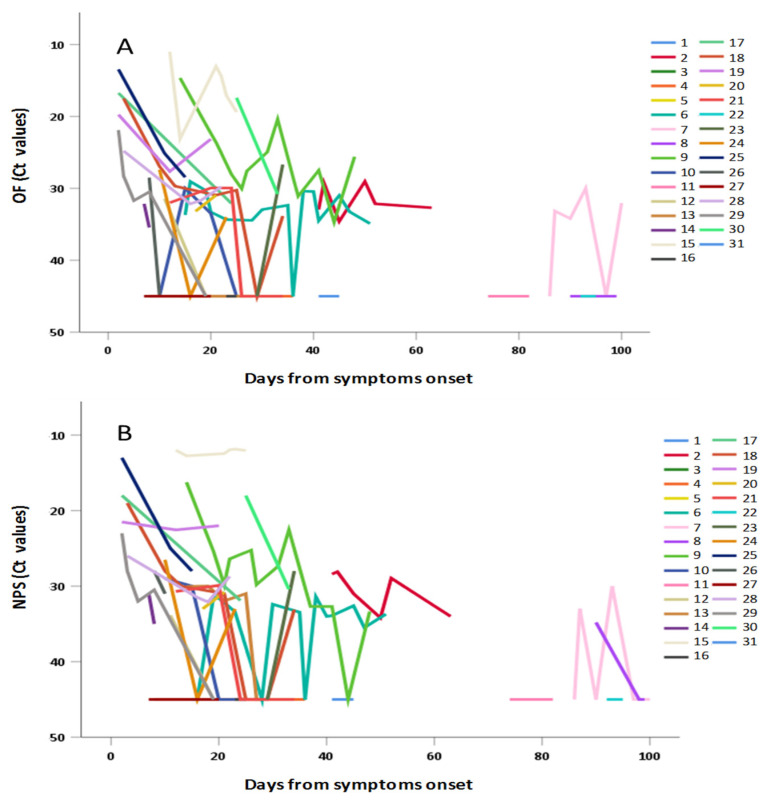
Shedding of SARS-CoV-2 RNA in (**A**) OF and (**B**) NPS samples from individuals with repeated measures, according to days from symptoms.

The data obtained in individuals with repeated measures have been separately shown in Figure 4A: OF and Figure 4B: NPS confirming in both matrices the general trend to progressive decrease of viral RNA concentration (i.e., increase of Ct values) observed in Figure 3 and in Table 2.

### 3.5. Sub-Group Analyses for OF vs. NPS vs. BAL Samples

**Figure 5:**
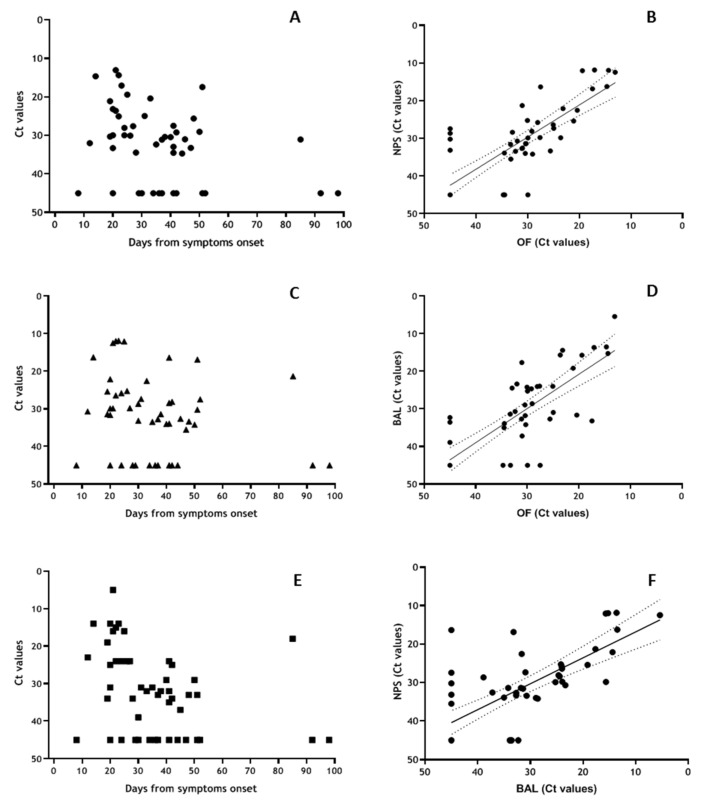
Shedding of SARS-CoV-2 RNA in OF, NPS and BAL samples based on data symptoms onset and correlation analyses. Results obtained from 50 samples coming from 12 patients with severe COVID-19 disease in the Intensive Care Unit, showing more severe presentation (PaO2/FiO2 < 100) tested for the presence of SARS-CoV-2 RNA in OF ((**A**), round symbol), NPS ((**C**), triangular symbol) and BAL ((**E**), square symbol) and expressed as Ct values vs. days from symptoms onset. Correlation analysis from Ct values obtained from NPS vs. OF (**B**), BAL vs. OF (**D**) and NPS vs. BAL (**F**).

Analyses of a subgroup of severe patients for whom repeated parallel NPS, OF and BAL samples were available (Figure 5) showed 78% positivity (Ct values < 40) in all district during the first 30 DSO; 70% positivity in OF, 74% in NPS and 65% in BAL between 30 and 60 DSO and 33% of positivity in all matrices > 60 DSO (Figure 5A,C,E). Elevated concordance was observed for virus detection in the various matrices (NPS vs. OF: κ = 0.848, 95% CI = 0.684–1.00; BAL vs. OF: κ = 0.714, 95% CI = 0.501–0.927; OF vs. BAL: κ = 0.646, 95% CI = 0.489–0.883), and highly significant correlation between the Ct values obtained on the three matrices (NPS vs. OF: r = 0.810, *p* < 0.001; BAL vs. OF: r = 0.797, *p* < 0.001; NPS vs. BAL: r = 0.732, *p* < 0.001) (Figure 5B,D,F). Statistical comparison between OF, NPS and BAL has been performed, showing no significant difference between median Ct values in the three district, neither in total nor according to different DSO intervals (Table 4). Nevertheless, when considering the earlier time interval (0–30 DSO), median Ct values in BALwere lower respect to NPS and OF, although the difference was not statistically significant (*p* > 0.05).

**Figure 6:**
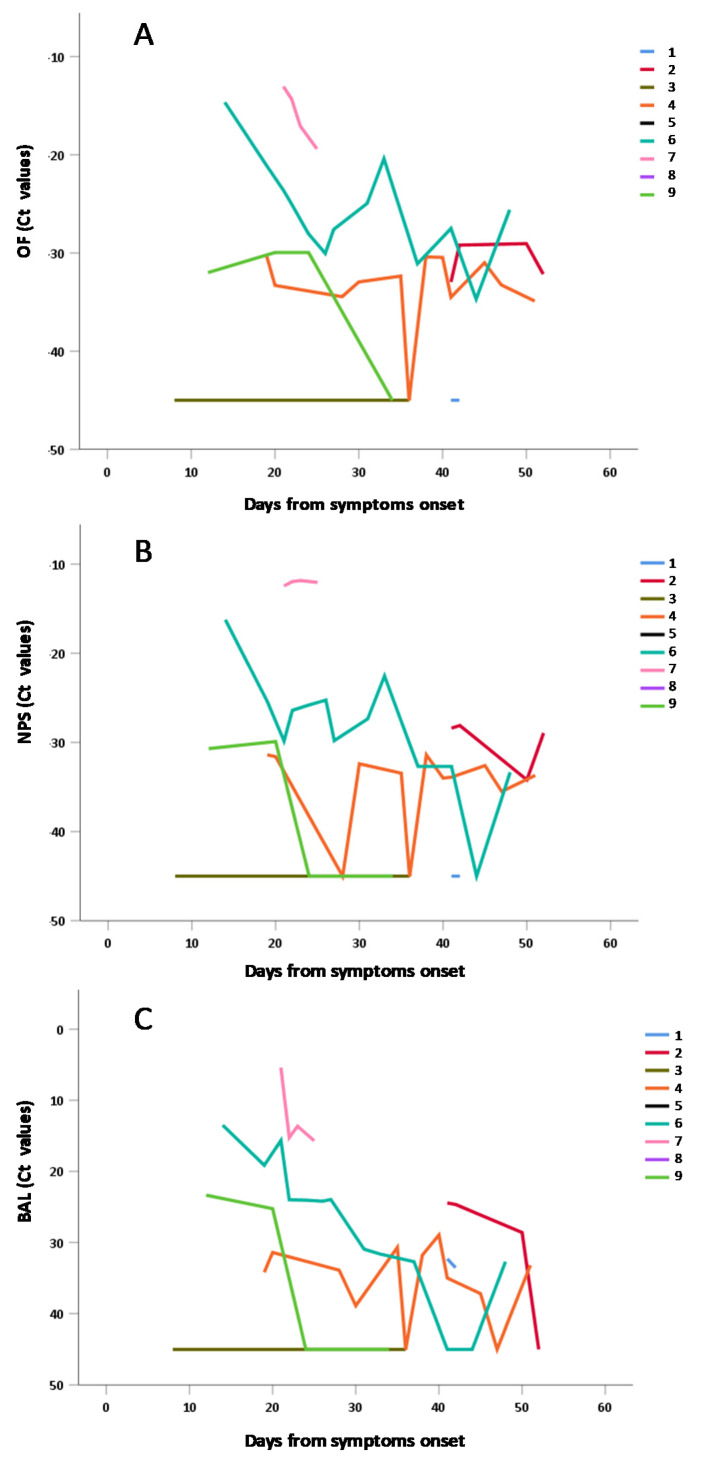
Shedding of SARS-CoV-2 RNA in OF (**A**), NPS (**B**) and BAL (**C**) NPS samples from individuals with repeated measures, according to days from symptoms.

We re-analyzed data of Figure 5A,C,E only for individual repeated measures, in order to show interpersonal and intrapersonal variability (Figure 6).

The data obtained in individuals with repeated measures in the three matrices have been separately shown in Figure 6A: OF; Figure 6B: NPS; Figure 6C: BAL confirming in both matrices the general trend to progressive decrease of viral RNA concentration (i.e., increase of Ct values) observed in Figure 5 and in Table 4.

## 4. Discussion

There are three important findings from our study. First, our results indicate that Simplexa™ COVID-19 Direct assay applied to OF has high analytical sensitivity and specificity, similar to that observed for NPS; in addition the rate of detection of SARS-CoV-2 in OF by the Simplexa™ COVID-19 Direct assay is similar to that of a standard test, based on Corman’s protocol, and Ct values from both tests are highly correlated.

Second, results from testing on paired OF, NPS and BAL samples by Simplexa™ COVID-19 Direct assay showed almost perfect concordance for virus detection, and high correlation of Ct values. Third, this assay detected prolonged oral shedding of SARS-CoV-2 60 DSO, which continued at least as long as nasopharyngeal shedding did. Hence, from these results, if appears that the use of this commercial assay to detect SARS-CoV-2 RNA in OF is of potentially high clinical utility for diagnosis and virological monitoring purposes.

Key advantages of the Simplexa™ COVID-19 Direct assay are simple operation procedures, with an all-in-one reagent mix and high-speed of detection in just over an hour, which is significantly faster than the up to seven hours required by traditional extraction followed by amplification technologies, currently used to detect SARS-CoV-2 RNA in OF samples [11,12,13,14,15,16,18]. Moreover, the test does not require extra-equipment (i.e centrifuges or an extraction system) and technical laboratory infrastructure, being suitable for the field settings and for near-to-patient diagnosis. The only limitation of the assay is the small number of samples which can be tested in a run, since each instrument can support a ring of maximum eight position.

Several recent studies have showed that OF could be an appropriate sample for diagnosis of SARS-CoV-2 [6,22]. The meta-analysis by Czumbel et al. on the reliability and consistency of SARS-CoV-2 viral RNA detection in OF specimens found 91% (95%CI = 80%–99%) sensitivity for OF tests and 98% (95%CI 89%–100%) sensitivity for NPS in previously confirmed COVID-19 infected patients [22].

Diagnostic testing for SARS-COV-2 RNA detection in clinical specimens supports decision making for clinical, infection control or public health management. SARS-CoV-2 detection is essential for patient care, triage and isolation in healthcare facilities. OF specimen offers an option for self-sampling, especially in situations where other specimens are difficult to obtain.

SARS-CoV-2 RNA detection in OF can also be used for screening of close contacts for asymptomatic infection and disease as part of contact tracing or outbreak investigations, local surveillance programmes and for screening specific groups like healthcare and social workers. It could also be useful for early control of viral transmission to vulnerable persons living in closed institutions and long-term care facilities. Apart from real-time use for medical or public health case management and transmission control, tests using OF as a specimen for virus detection can be used to surveillance and determining incidence and prevalence of infection and disease.

In a situation where NPS or other above mentioned specimen is not acceptable, OF could be considered a valuable alternative specimen. On 8th May, 2020, the U.S. Food and Drug Administration had authorized the first diagnostic test with the option of using home-collected OF samples for COVID-19 testing issuing an emergency use authorization (/media/137773/download) (EUA) to Rutgers Clinical Genomics Laboratory for their COVID-19 laboratory developed test. The Simplexa™ COVID-19 Direct assay on OF to detect SARS-CoV-2 RNA has high sensitivity, and provides an additional alternative for diagnosis and monitoring SARS-CoV-2 shedding. Further evaluation of the Simplexa™ COVID-19 Direct assay for home based self-use, surveillance purposes to monitor the epidemiologic situation in terms of incidence and prevalence of infection and disease in the community are required.

## Figures and Tables

**Table 1:** Patient’s number and characteristics.

	Asymptomatic	Paucisymptomatic	Severe (PaO2/FiO2 < 100)	Negative	Tot
**Patients (N°)**	14	61	12	77	164
**Samples (N°)**	18	154	50	115	337

**Table 2:** Comparison of median Ct values in OF and NPS, total, and according with DSO.

		OF: Median Ct Values (Range)	NPS: Median Ct Values (Range)
	**Tot**	32.2 (11.0–45)	32.0 (11.9–45)
**DSO**	**0–30**	31.0 (11.0–45)	31.0 (11.9–45)
	**31–60**	33.3 (20.4–45)	33.4 (22.6–45)
	**>60**	45.0 (30.0–45)	45.0 (21.3–45)

**Table 3:** Comparison of median Ct values in OF and NPS according to age.

	Age		Significance (*p* Value)
	<60 Years	>60 Years	
**OF: Median Ct values (range)**	29.9 (13.5–45)	34.3 (11.0–45)	*p* = 0.0007
**NPS: Median Ct values (range)**	29.8 (13.0–45)	33.9 (11.9–45)	*p* = 0.0004

**Table 4:** Comparison of median Ct values in OF, NPS and BAL, total and according with DSO.

		OF: Median Ct Values (Range)	NPS: Median Ct Values (Range)	BAL: Median Ct Values (Range)
	**Tot**	31.0 (13.1–45)	31.4 (5–45)	32.5 (11.9–45)
**DSO**	**0–30**	30.0 (13.1–45)	29.8 (11.9–45)	24.1 (5.0–45)
	**31–60**	32.7 (17.5–45)	33.4 (16.4–45)	33.4 (24.5–45)
	**>60**	45 (31.1–45)	45 (21.3–45)	45 (17.7–45)
